# Artificial intelligence-based models in predicting acute exacerbations and diagnosing pediatric asthma: a systematic review and meta-analysis

**DOI:** 10.1186/s12911-026-03599-7

**Published:** 2026-05-29

**Authors:** Qingxia Shi, Lu Zhu, Wenwen Wang, Xuemei Li

**Affiliations:** 1 Department of Pediatrics, Chongqing Health Center for Women and Children, Chongqing, 400000 China; 2 Department of Pediatrics, Women and Children’s Hospital of Chongqing Medical University, Chongqing, 400000 China; 3 NHC Key Laboratory of Birth Defects and Reproductive Health, Chongqing, 400000 China; 4 Chongqing Research Center for Prevention & Control of Maternal and Child Diseases and Public Health, Chongqing, 400000 China

**Keywords:** Artificial intelligence, Asthma, Children, Asthma exacerbations, Meta-analysis

## Abstract

**Purpose:**

This systematic review and meta-analysis evaluated the performance of artificial intelligence (AI)-based models in diagnosing pediatric asthma and predicting acute asthma exacerbations.

**Methods:**

A comprehensive literature search was conducted across PubMed, Embase, and Web of Science. The initial search was conducted up to December 6, 2024, and a supplementary search was conducted in April 2025 to identify newly published or newly indexed studies. Diagnostic performance metrics, including sensitivity, specificity, area under the curve (AUC), and 2 × 2 diagnostic data, were extracted or reconstructed. A bivariate random-effects model was used for meta-analysis, and study quality was assessed using a modified QUADAS-2 tool with PROBAST-informed signaling questions.

**Results:**

A total of 18 studies were included: 13 studies evaluated AI-based models for pediatric asthma diagnosis and five studies evaluated AI-based models for predicting acute asthma exacerbations. For asthma diagnosis, internal validation showed a sensitivity of 0.87, specificity of 0.93, and AUC of 0.96, while external validation showed a sensitivity of 0.81 and specificity of 0.94. For acute exacerbation prediction, internal validation showed a sensitivity of 0.59, specificity of 0.79, and AUC of 0.68. These estimates should be interpreted cautiously because heterogeneity was very high and external validation evidence was limited.

**Conclusion:**

AI-based models showed promising but preliminary diagnostic performance for pediatric asthma, whereas their performance for predicting acute exacerbations remained limited. The findings should be interpreted cautiously because of substantial heterogeneity, potential publication bias, and limited external validation. Future studies should use standardized definitions and independent external validation before these models are implemented in clinical practice.

**Clinical trial number:**

Not applicable.

**Supplementary Information:**

The online version contains supplementary material available at 10.1186/s12911-026-03599-7.

## Introduction

Asthma in pediatric patients is a long-lasting respiratory condition marked by inflammation of the airways, increased sensitivity, and reversible blockage of airflow [1]. This condition affects approximately 1,030.3 individuals per 100,000 people globally [2]. It stands as the most prevalent chronic respiratory illness among children, with symptoms frequently appearing in early childhood, although they do not always continue into the school years [3]. Asthma exacerbations cause sudden symptom worsening, which significantly impact healthcare utilization, school absenteeism, and quality of life [4]. Diagnosing pediatric asthma is challenging, particularly in preschool-age children, as symptoms such as wheezing are often transient and may resolve spontaneously [5]. Accurate diagnosis and prediction of exacerbations are essential to avoid underdiagnosis and overtreatment, which are common issues in this age group [6].

Traditional diagnostic approaches for pediatric asthma rely on symptoms, physical findings, pulmonary function tests (PFTs), and related clinical information. However, young children may not perform PFTs reliably, and diagnostic criteria vary across clinical settings and studies, which can complicate accurate diagnosis and may contribute to heterogeneity in model evaluation [7, 8]. Artificial intelligence (AI), including machine learning (ML) and deep learning (DL), has increasingly been used to support pediatric asthma diagnosis and risk prediction [9, 10]. These methods can identify patterns in clinical, physiological, imaging, genomic, and electronic health record data; however, their clinical value depends on transparent model development, reliable validation, and standardized outcome definitions [11, 12].

To address these challenges, this systematic review and meta-analysis aimed to evaluate the performance of AI-based models in diagnosing pediatric asthma and predicting acute asthma exacerbations.

## Methods

The protocol of our systematic review was registered in PROSPERO (CRD420251135350). The meta-analysis was conducted in accordance with the Preferred Reporting Items for a Systematic Review and Meta-analysis of Diagnostic Test Accuracy Studies (PRISMA-DTA) statement [13], and the PRISMA-DTA checklist was provided as Supplementary Table 1.

### Search strategy

The literature search was performed independently by two investigators across PubMed, Embase, and Web of Science. The initial search deadline was December 6, 2024, and a supplementary search was conducted in April 2025 to identify newly published or newly indexed studies; therefore, April 2025 represented the final search update. The search strategy incorporated terms for artificial intelligence, asthma, pediatric populations, and diagnostic or predictive performance. Detail strategy was shown in Supplementary Table 2.

### Inclusion and exclusion criteria

The research was selected using the PICOS framework. Population (P): children and adolescents aged 0–17 years with asthma or at risk of asthma-related acute exacerbations. Intervention (I): AI-based predictive or diagnostic models, including ML algorithms and DL models such as convolutional neural networks. Comparator (C): no comparator was required. Outcomes (O): sensitivity, specificity, AUC, and 2 × 2 diagnostic data. Study design (S): original studies evaluating AI algorithms focused on asthma diagnosis or asthma exacerbation prediction.

We excluded animal studies, reviews, case reports, conference abstracts, meta-analyses, editorial letters, non-AI studies, studies outside the research scope, studies without sufficient data to derive true positive (TP), true negative (TN), false negative (FN) and false positive (FP), and studies in which the AI algorithm primarily targeted respiratory diseases other than asthma or asthma exacerbations. Studies were excluded if the AI algorithm primarily targeted respiratory diseases other than asthma or asthma exacerbations.

### Quality assessment

To assess study quality, we used a modified QUADAS-2 tool [14] and added prediction model risk of bias assessment tool (PROBAST)-informed signaling questions relevant to prediction-model bias [15], including validation method, predictor-reference overlap, model selection, and analysis (Supplementary Tables 3–4). The modified tool itself is provided in the revised supplementary material. The revised QUADAS-2 tool covers four domains: patient selection, index test (AI algorithms), reference standard, and analysis. Risk of bias was assessed in all four domains, and applicability concerns were assessed in patient selection, index test, and reference standard domains. Any discrepancies in study quality assessment were resolved through discussion between the two reviewers. Inter-rater agreement was assessed using Cohen’s kappa, which showed substantial agreement (κ = 0.81).

### Data extraction

Two reviewers (QS and LZ) independently screened titles and abstracts, assessed eligibility, and extracted data. Disagreements were resolved through discussion with a third reviewer (WW). Any discrepancies in data extraction were resolved through discussion between the two reviewers. Inter-rater agreement was assessed using Cohen’s kappa, which showed almost perfect agreement (κ = 0.89). Extracted items included first author, publication year, country, study design, reference standard, target condition, validation setting, AI method, optimal algorithm, sample size, and TP, TN, FP, and FN.

When TP, FP, FN, and TN were directly available, these values were extracted. When 2 × 2 data were unavailable, we reconstructed the table using reported sensitivity, specificity, the number of reference-standard-positive participants, total sample size, and, when applicable, the operating point corresponding to the highest Youden index on the ROC curve.

When multiple models were reported, we selected the model prespecified or recommended by the original study; when no primary model was specified, the model with the best validation AUC was extracted. This approach may overestimate performance and was therefore acknowledged as a limitation.

### Outcome measures

Key outcome measures include sensitivity, specificity, and AUC of both internal and external validation sets in childhood asthma diagnosis and asthma exacerbation prediction. Sensitivity assesses the probability that an AI model will correctly identify a real asthma patient or an acute asthma attack. The calculation formula is TP/ (TP + FN). Specificity indicates the likelihood that an AI model accurately identifies a patient who does not have asthma or an acute episode of non-asthma. The calculation formula is TN/ (TN + FP). The AUC, serves as an all-encompassing metric for evaluating the model’s performance in differentiating between positive and negative instances.

Internal validation was defined as validation performed within the same study population, data source, or development cohort, including hold-out testing, cross-validation, or apparent/internal test-set evaluation as reported by the original study. External validation was defined as validation in an independent population, site, cohort, or database.

### Statistical analysis

A bivariate random-effects model was used to assess diagnostic and predictive performance. Forest plots and summary receiver operating characteristic curves were used to display pooled sensitivity, specificity, AUC, 95% confidence intervals, and prediction intervals. Heterogeneity was assessed using Higgins I^2^ statistics. To explore potential sources of heterogeneity, we conducted subgroup analyses and meta-regression. For publication-bias assessment in the diagnostic accuracy analysis, Deeks’ funnel plot asymmetry test was selected because it has been evaluated for diagnostic test accuracy reviews and is recommended for assessing funnel plot asymmetry and small-study effects in this context [16, 17]. This test was applied only to the asthma diagnosis analysis, for which at least ten studies were available, and was not applied to acute exacerbation prediction because fewer than ten studies were available. We did not apply the trim-and-fill method because non-DTA funnel-plot adjustment methods have not been validated for diagnostic accuracy meta-analyses [17].

## Results

### Study selection

**Fig. 1:**
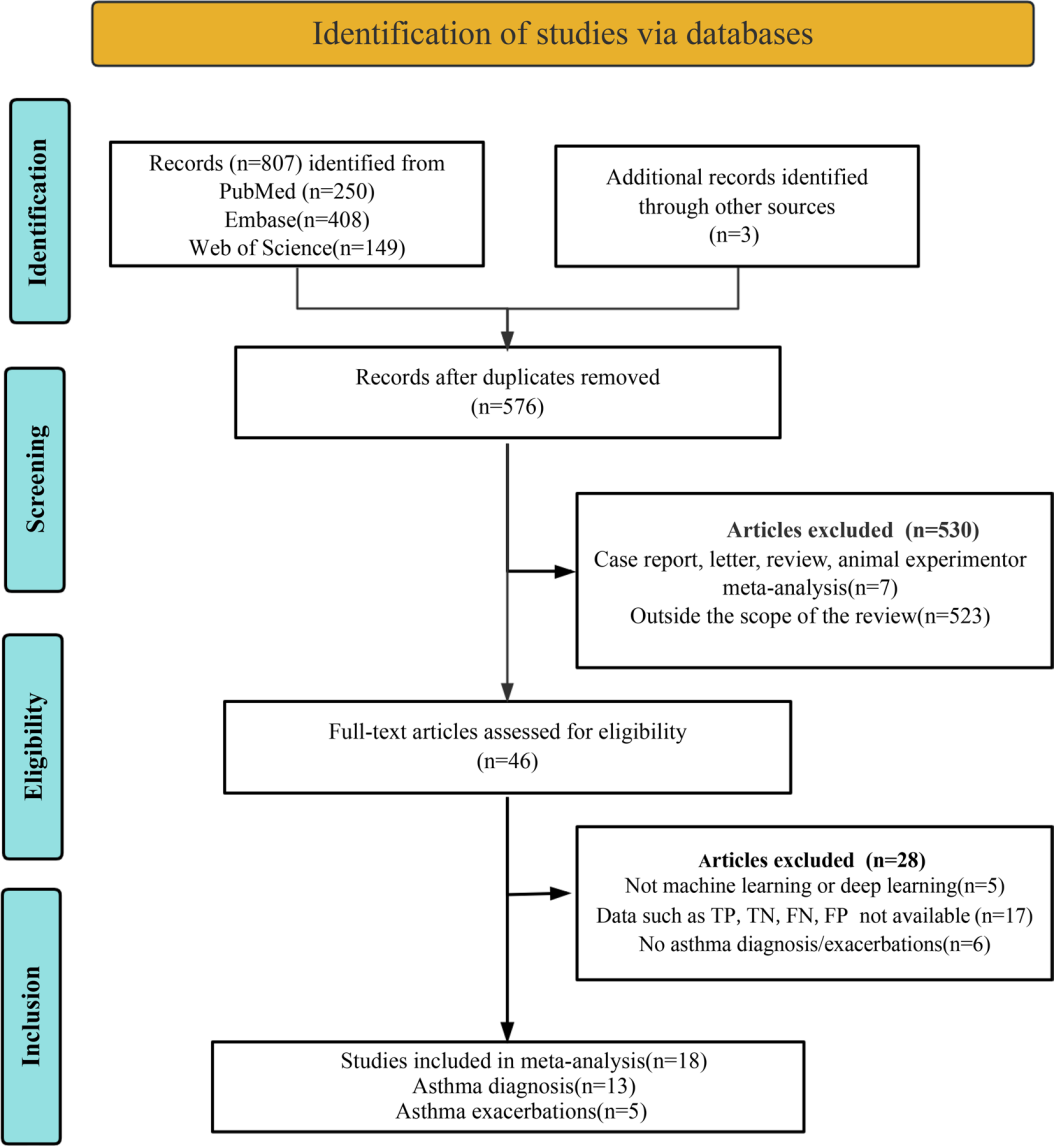
The revised PRISMA flow diagram depicts the systematic process of study selection

The database search identified 807 records, and three additional records were identified through other sources, yielding 810 records in total. After 234 duplicate records were removed, 576 unique records were screened. During title and abstract screening, 530 records were excluded, including seven case reports, letters, reviews, animal experiments, or meta-analyses and 523 records outside the scope of the review. After full-text review, 28 studies were excluded because they did not use ML or DL (*n* = 5), lacked sufficient data such as TP, TN, FN, and FP (*n* = 17), or did not evaluate asthma diagnosis or asthma exacerbations (*n* = 6). Finally, 18 studies met the inclusion criteria (Fig. 1).

### Study description and quality assessment

There were 18 studies, of which 13 focused on asthma diagnosis [18–30] and five examined asthma exacerbations [31–35]. For the diagnosis of asthma, the internal validation set included 13 studies [18–30], with a total of 206,469 patients (range: 41–195,666), and the external validation included two studies [23, 30] with a total of 2,405 patients (range: 282-2,123). The study was published from 2004 to 2024. In the meta-analysis, eight of the studies [18, 20–22, 24, 25, 29, 30] were identified as retrospective, while five studies [19, 23, 26–28] were classified as prospective. The most commonly used modeling methods are random forest (3/13) [19, 20, 26], support vector machine (2/13) [23, 24], and artificial neural network (2/13) [25, 29]. For asthma exacerbations, the internal validation set included four studies [32–35], with a total of 40,195 patients (range: 417 − 22,631), and external validation included three studies [31, 32, 35] with a total of 1,559 patients (range:82 − 1,313). The study was published from 2011 to 2024. Among the studies analyzed in the meta-analysis, three [32–34] were conducted retrospectively, while two [31, 35] were prospective studies. Tables 1 and 2, and 3 presented a summary of the study along with patient and technical characteristics.

**Table 1:** Study and patient characteristics of the included studies

Author	Year	Country	Study design	Reference standard	Age	Target	No. of total patients			No. of asthma/asthma exacerbations patients		
							Training	Internal validation	External validation	Training	Internal validation	External validation
Afzal et al.	2013	Netherlands	Retro	at least one entry in their medical record containing an asthma diagnosis confirmed by a specialist	aged 5–18 years	asthma diagnosis	5032	5032	NA	308	308	NA
Ahmadiankalati et al.	2024	Canada	Pro	parent-reported more than 2 episodes of wheezing within the last year	before 3 years of age	asthma diagnosis	1956	838	NA	386	46	NA
Dexheimer et al.	2007	USA	Retro	based on free-text terms: “asthma exacerbation”, “status asthmaticus”, “wheezing”, or “reactive airway disease”, confirmed by board-certified physicians through manual chart review	2 to 18 years old	asthma diagnosis	3017	1006	NA	289	96	NA
Filippo et al.	2024	Italy	Retro	age 6–17 years and confirmed diagnosis of SA according to ERS/ATS guidelines	age 6–17 years	asthma diagnosis	41	41	NA	20	20	NA
Hamad et al.	2023	Canada	Retro	the Canadian Chronic Disease Surveillance System	age 1-18years	asthma diagnosis	195,666	195,666	NA	34,565	34,565	NA
Kothalawala et al.	2021	UK	Pro	a doctor diagnosis of asthma ever and at least one episode of wheezing or use of asthma medication in the last 12 months	age 0-5years	asthma diagnosis	365	183	282	51	25	33
Maduko et al.	2007	Caucasian and Hispanic	Retro	NA	NA	asthma diagnosis	112	112	NA	30	30	NA
Pooja et al.	2024	Austria	Retro	The data included bronchial tube disease status as a key parameter for confirming asthma diagnosis, along with PEF measurements and spirometry readings of major pulmonary functions.	NA	asthma diagnosis	326	326	NA	163	163	NA
Raciborski et al.	2018	Poland	Pro	(1) Questionnaire survey (questionnaire in electronic form; (2) Interview and physical examination; (3) SPT—outpatient examination; (4) Spirometry with reversibility test—outpatient examination	age 6-18years	asthma diagnosis	1008	1008	NA	113	113	NA
Salih et al.	2024	Iraq	Pro	(1) Family history; (2) Eczema; (3) Cough; (4) no Headache; (5) Temperature normal; (6) no Chills	below 6 years	asthma diagnosis	358	154	NA	173	75	NA
Sanders et al.	2006	USA	Pro	asthma exacerbation, status asthmaticus, wheezing, or reactive airway disease, as well as cases where asthma was considered but ruled out after beta-agonist medication trial	age 2-18years	asthma diagnosis	3017	1006	NA	290	95	NA
Tomita et al.	2004	Japan	Retro	NA	NA	asthma diagnosis	344	344	NA	172	172	NA
Yu et al.	2020	China	Retro	Children’s bronchial asthma diagnosis and prevention (2016 version)	under the age of 14	asthma diagnosis	3008	753	2123	1299	325	337
Farion et al.	2013	Canada	Pro	ED length of stay greater than 4 h or requiring admission	2 to 17 years	asthma exacerbations	240	NA	82	131	NA	56
Gorham et al.	2023	USA	Retro	ED visit for asthma within one year of primary care encounter	aged 2–18 years	asthma exacerbations	26,008	8634	1313	7708	502	98
Rezaeiahari et al.	2024	USA	Retro	ED visit and/or hospitalization	aged 5–18 years	asthma exacerbations	22,631	22,631	NA	2042	2042	NA
Wang et al.	2019	USA	Retro	ED visit in the following 3 months	aged between 6 months and 18 years	asthma exacerbations	19,865	8513	NA	661	190	NA
Xu et al.	2011	USA	Pro	ED visits or hospitalizations	NA	asthma exacerbations	417	417	164	127	127	50

Retro Retrospective; Pro Prospective; NA Not available; SA Severe asthma; ERS/ATS European respiratory society/American thoracic society; PEF Peak expiratory flow; SPT Skin prick test; ED Emergency department

**Table 2:** Technical aspects of included studies on childhood asthma diagnosis

Author	Year	AI method	Optimal AI algorithms ^a^	Internal validation sets				External validation sets			
				TP	FP	FN	TN	TP	FP	FN	TN
Afzal et al.	2013	ML	RIPPER	302	236	6	4488	NA	NA	NA	NA
Ahmadiankalati et al.	2024	ML	Random forest	3	0	43	793	NA	NA	NA	NA
Dexheimer et al.	2007	ML	MMHC	86	90	10	820	NA	NA	NA	NA
Filippo et al.	2024	ML	Random forest	19	1	1	20	NA	NA	NA	NA
Hamad et al.	2023	ML	LASSO	24,887	49,941	9678	111,160	NA	NA	NA	NA
Kothalawala et al.	2021	ML	SVM	18	19	7	139	18	22	15	227
Maduko et al.	2007	ML	ANN	30	3	0	79	NA	NA	NA	NA
Pooja et al.	2024	ML	SVM	136	27	27	136	NA	NA	NA	NA
Raciborski et al.	2018	ML	Random forest	95	79	18	816	NA	NA	NA	NA
Salih et al.	2024	DL	2-1D-CNNs	74	0	1	79	NA	NA	NA	NA
Sanders et al.	2006	ML	Bayesian network	86	105	9	806	NA	NA	NA	NA
Tomita et al.	2004	ML	ANN	128	48	44	124	NA	NA	NA	NA
Yu et al.	2020	ML	CatBoost	262	66	63	362	312	86	25	1700

AI Artificial Intelligence; ML Machine learning; DL Deep learning; TP True positive; TN True negative; FP False positive; FN False negative; NA Not available; RIPPRE Repeated incremental pruning to produce error reduction; MMHC Max-min hill-climbing; LASSO Least absolute shrinkage and selection operator; SVM Support vector machine; ANN Artificial neural network; 2-1D-CNNs Two one-dimensional convolutional neural networks; CatBoost Categorical boosting

^a^ The term Optimal AI algorithm refers to the algorithm with the highest AUC value. AUC Area under the curve

**Table 3:** Technical aspects of included studies on asthma exacerbations in children

Author	Year	AI method	Optimal AI algorithms ^a^	Internal validation sets				External validation sets			
				TP	FP	FN	TN	TP	FP	FN	TN
Farion et al.	2013	ML	NB	NA	NA	NA	NA	39	7	17	19
Gorham et al.	2023	ML	LASSO	320	1862	182	6270	82	663	16	552
Rezaeiahari et al.	2024	ML	CRF	1123	4530	919	16,059	NA	NA	NA	NA
Wang et al.	2019	DL	ANN	97	688	93	7635	NA	NA	NA	NA
Xu et al.	2011	ML	RF	84	116	43	174	33	46	17	68

AI Artificial intelligence; ML Machine learning; DL Deep learning; TP True positive; TN True negative; FP False positive; FN False negative; NA Not available; NB Naive Bayes; LASSO Least absolute shrinkage and selection operator; CRF Conditional random forest; ANN Artificial neural networks; RF Random forests

^a^ The term Optimal AI algorithm refers to the algorithm with the highest AUC value. AUC Area under the curve

**Fig. 2:**
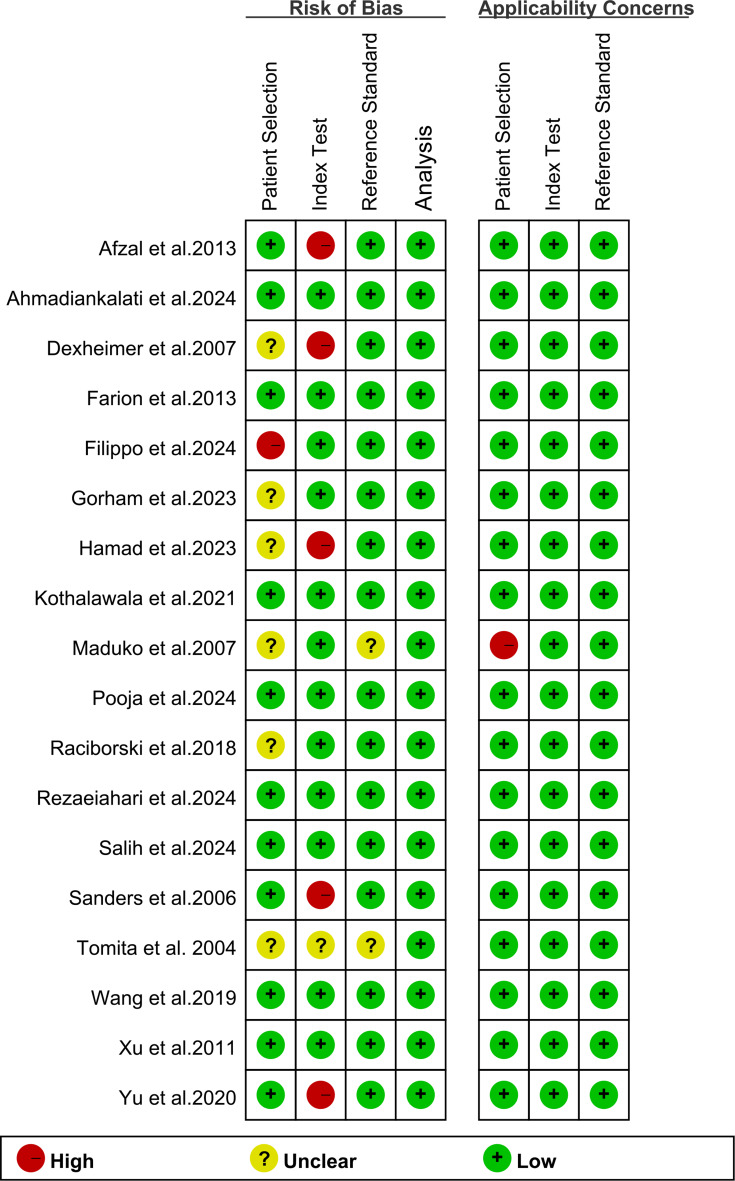
Risk of bias and applicability concerns in the included studies were assessed using the QUADAS-2 (Revised Quality Assessment of Diagnostic Accuracy Studies) tool

The risk of bias assessed using the revised QUADAS-2 instrument is illustrated in Fig. 2. One study was rated as high risk in patient selection because only patients with severe asthma were included [20], and one study had high applicability concern in patient selection because it included both childhood asthma and small airway disease [25]. Additionally, five studies [18, 21, 22, 28, 30] were classified as high risk in the index test (AI algorithms) domain due to predictor-reference standard overlap, introducing potential target leakage.

### Diagnostic performance of internal validation set for AI in diagnosing asthma and predicting acute asthma attacks

**Fig. 3:**
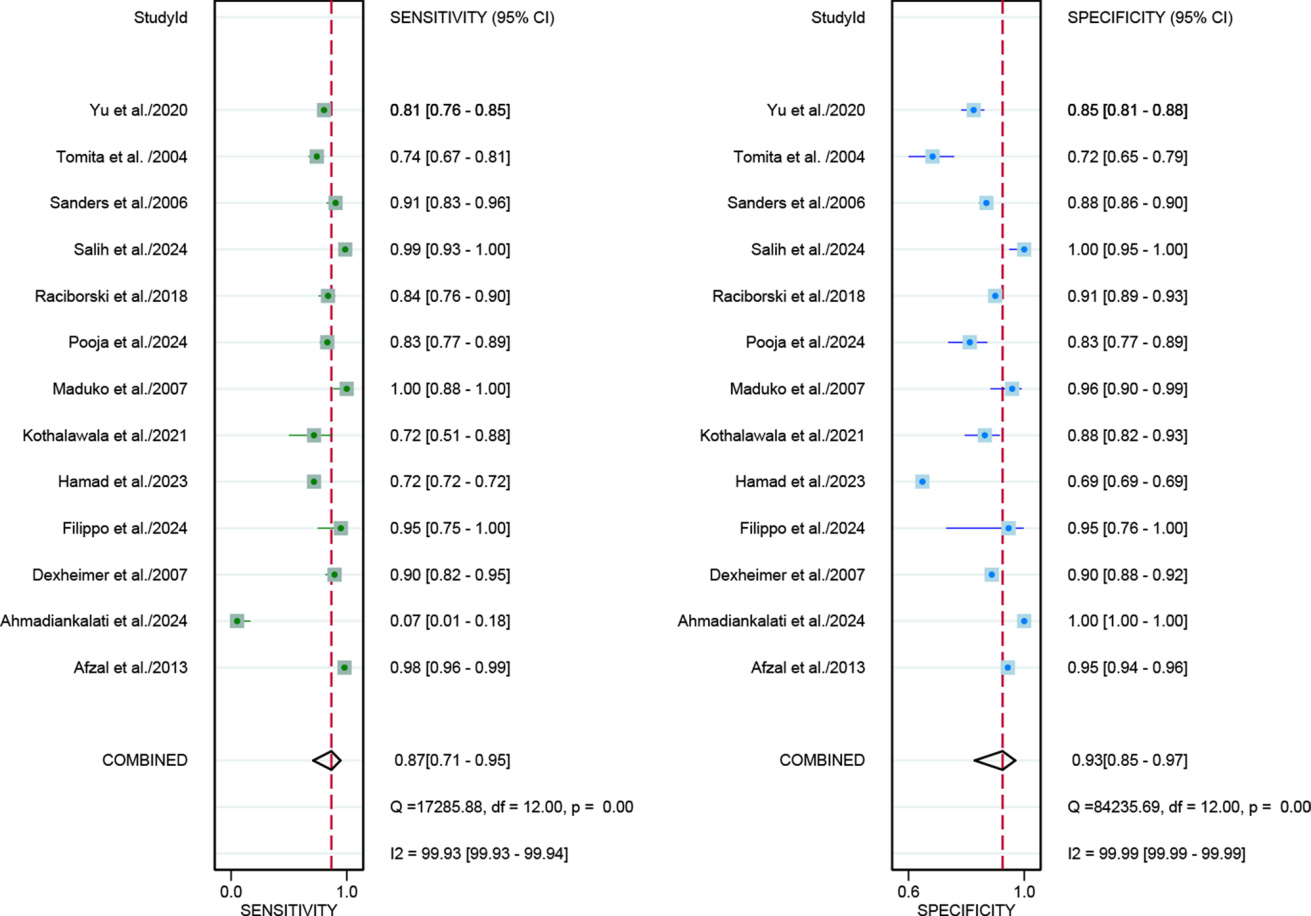
Forest plot of AI-based models for sensitivity and specificity in the diagnosis of pediatric asthma in internal validation. Squares represent sensitivity and specificity estimates from individual studies, with horizontal bars showing 95% confidence intervals. AI, artificial intelligence

**Fig. 4:**
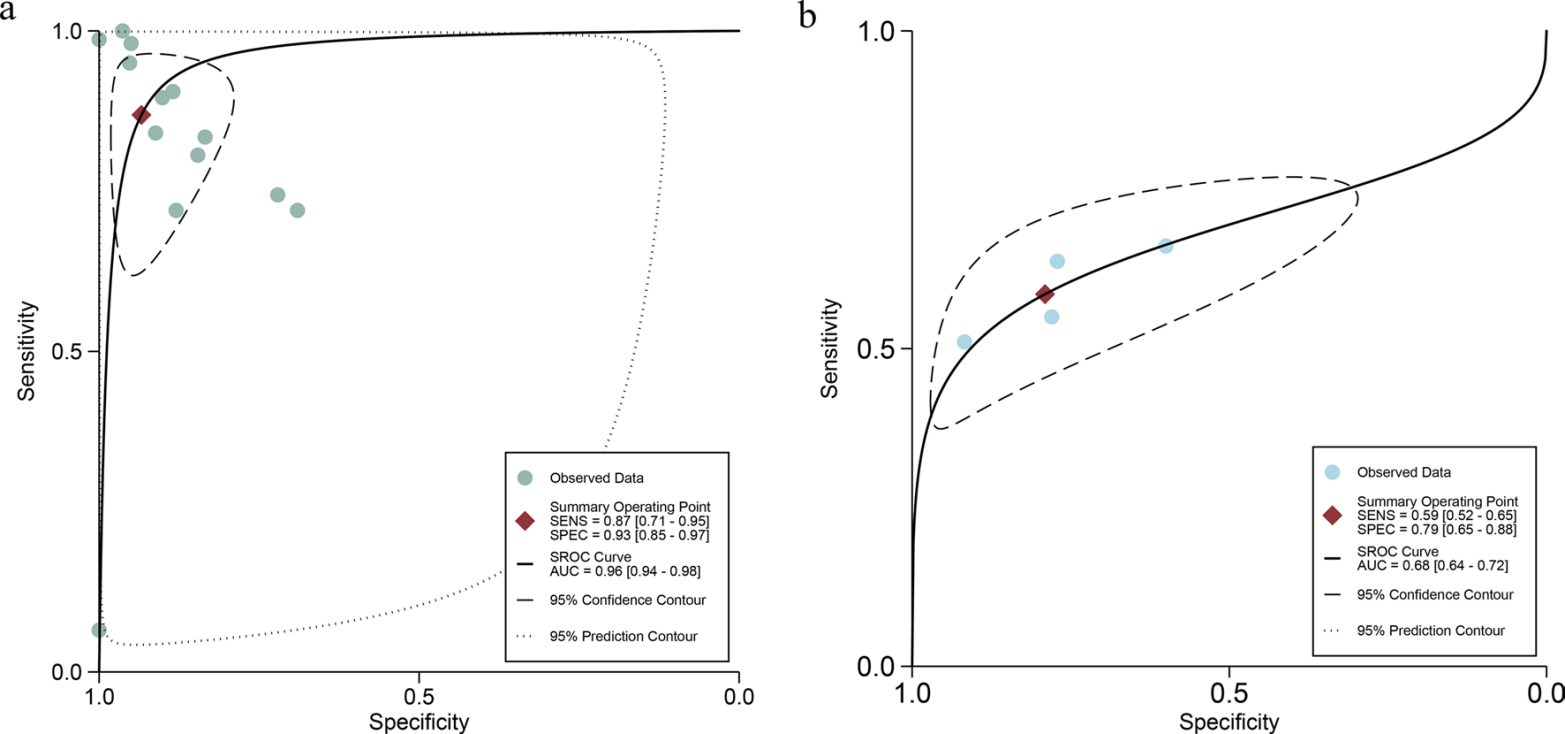
Summary receiver operating characteristic (SROC) curves evaluating AI-based models for (**a**) pediatric asthma diagnosis and (**b**) acute exacerbation prediction during internal validation. The diagnostic performance metrics of individual studies are represented by squares, with the solid line indicating the 95% confidence contour and dashed lines delineating the 95% prediction contour. AI, artificial intelligence

**Fig. 5:**
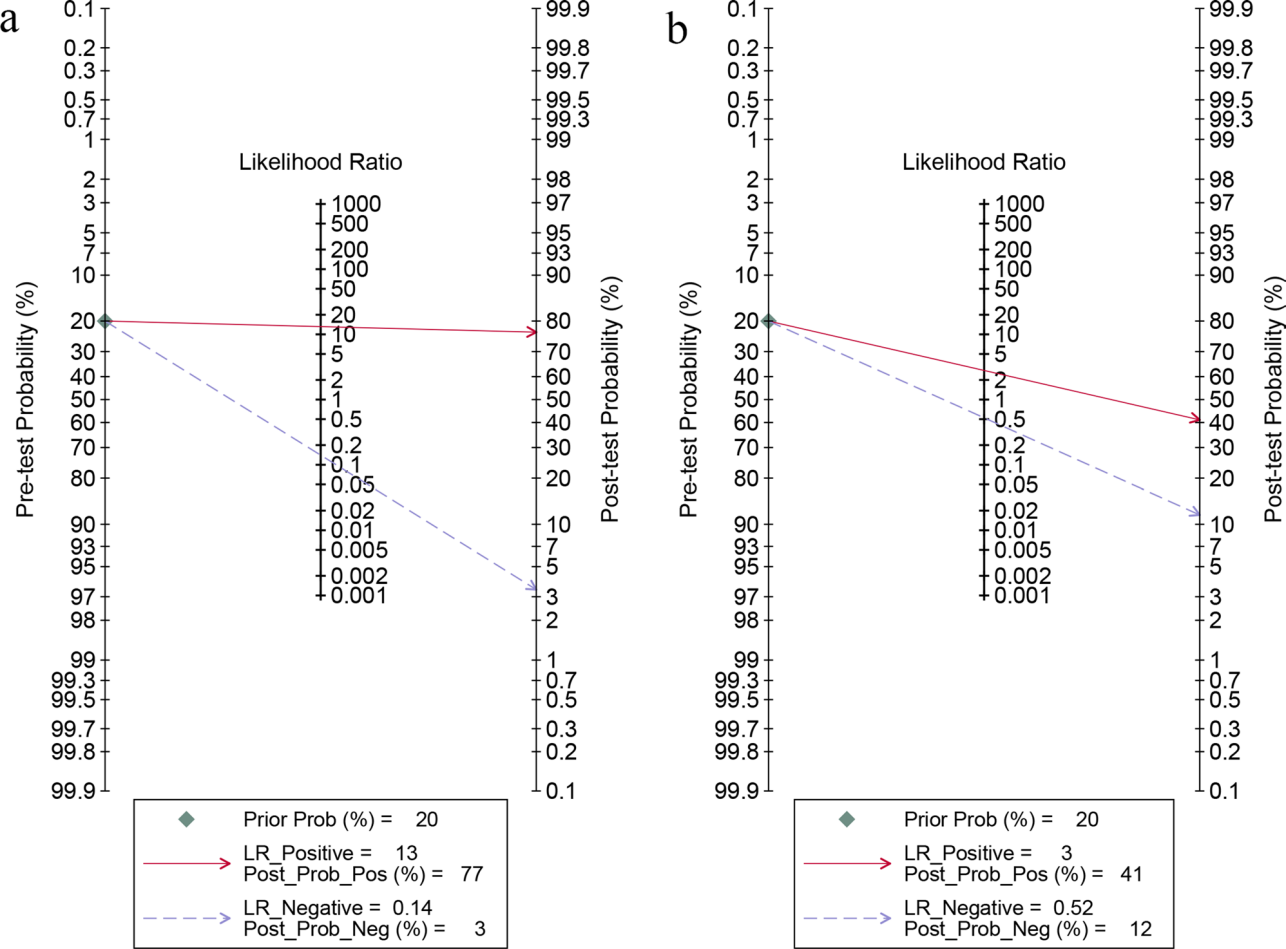
Fagan plot analysis evaluating AI model performance in (**a**) diagnosing pediatric asthma and (**b**) predicting acute exacerbations during internal validation. AI, artificial intelligence

For asthma diagnosis, the AI models had a sensitivity of 0.87 (95% CI: 0.71–0.95), specificity of 0.93 (95% CI: 0.85–0.97), and AUC of 0.96 (95% CI: 0.94–0.98) in internal validation (Figs. 3 and 4a). At a pre-test probability of 20%, the post-test probability after a positive diagnostic result was 77% and after a negative diagnostic result was 3%; the corresponding likelihood ratios were LR + = 13 and LR- = 0.14 (Fig. 5a).

**Fig. 6:**
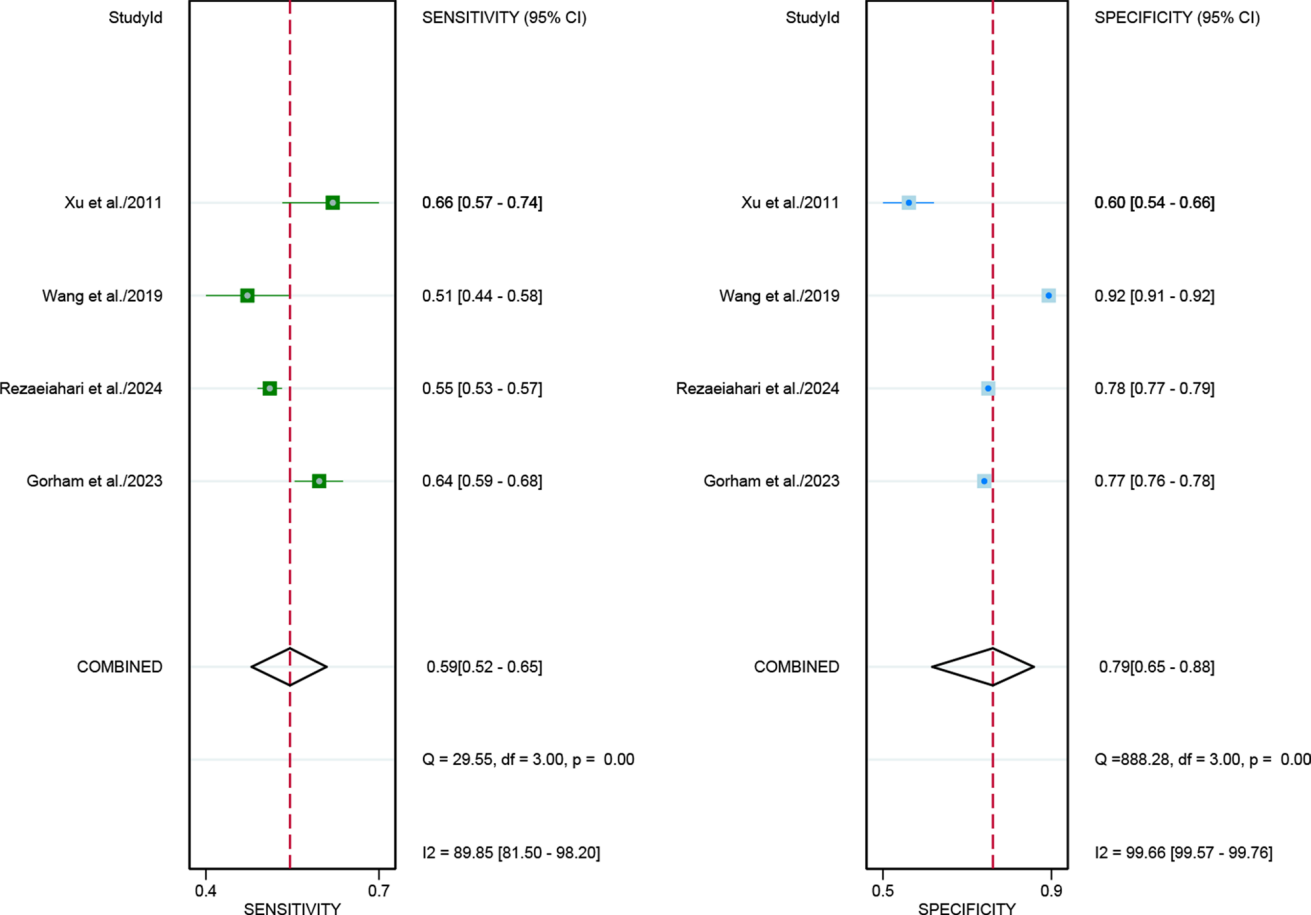
Forest plot of AI models for predicting acute exacerbations of asthma during internal validation. Data squares represent sensitivity and specificity estimates from individual studies, with error bars indicating 95% confidence intervals. AI, artificial intelligence

For acute asthma exacerbation prediction, the AI models had a sensitivity of 0.59 (95% CI: 0.52–0.65), specificity of 0.79 (95% CI: 0.65–0.88), and AUC of 0.68 (95% CI: 0.64–0.72) in internal validation (Figs. 4b and 6). At a pre-test probability of 20%, the post-test probability after a positive result was 41% and after a negative result was 12%; the corresponding likelihood ratios were LR + = 3 and LR- = 0.52 (Fig. 5b).

Substantial heterogeneity was observed in the internal validation set for asthma diagnosis (sensitivity I^2^ = 99.93%; specificity I^2^ = 99.99%). Meta-regression analysis indicated that the heterogeneity in specificity was associated with AI methods, whereas the heterogeneity in sensitivity was linked to data sources (Table 4).

**Table 4:** Subgroup analysis and meta-regression analysis on childhood asthma diagnosis

Covariate	Studies, *n*	Sensitivity (95%CI)	*P*-value	Specificity (95%CI)	*P*-value
**Study design**			0.29		0.21
Retrospective	8	0.91(0.81-1.00)		0.89(0.78–0.99)	
Prospective	5	0.78(0.53-1.00)		0.97(0.94-1.00)	
**Region**			0.56		0.99
Asian	3	0.90(0.71-1.00)		0.92(0.78-1.00)	
Non-Asian	10	0.86(0.73–0.99)		0.94(0.88-1.00)	
**AI method**			0.46		0.02
Deep learning	1	0.99(0.96-1.00)		1.00(1.00–1.00)	
Machine learning	12	0.84(0.72–0.96)		0.92(0.86–0.98)	
**Reconstructed data by ROC curve**			0.97		0.49
Yes	2	0.82(0.48-1.00)		0.84(0.56-1.00)	
No	11	0.88(0.76–0.99)		0.94(0.89-1.00)	
**Data sources**			< 0.001		0.80
EHR data	7	0.94(0.87-1.00)		0.93(0.83-1.00)	
Genomic data	2	0.32(0.13–0.78)		0.99(0.94-1.00)	

CI Confidence interval; AI Artificial intelligence; EHR Electronic Health Records

### Diagnostic performance of external validation sets for AI in diagnosing asthma and predicting acute asthma exacerbations

**Fig. 7:**
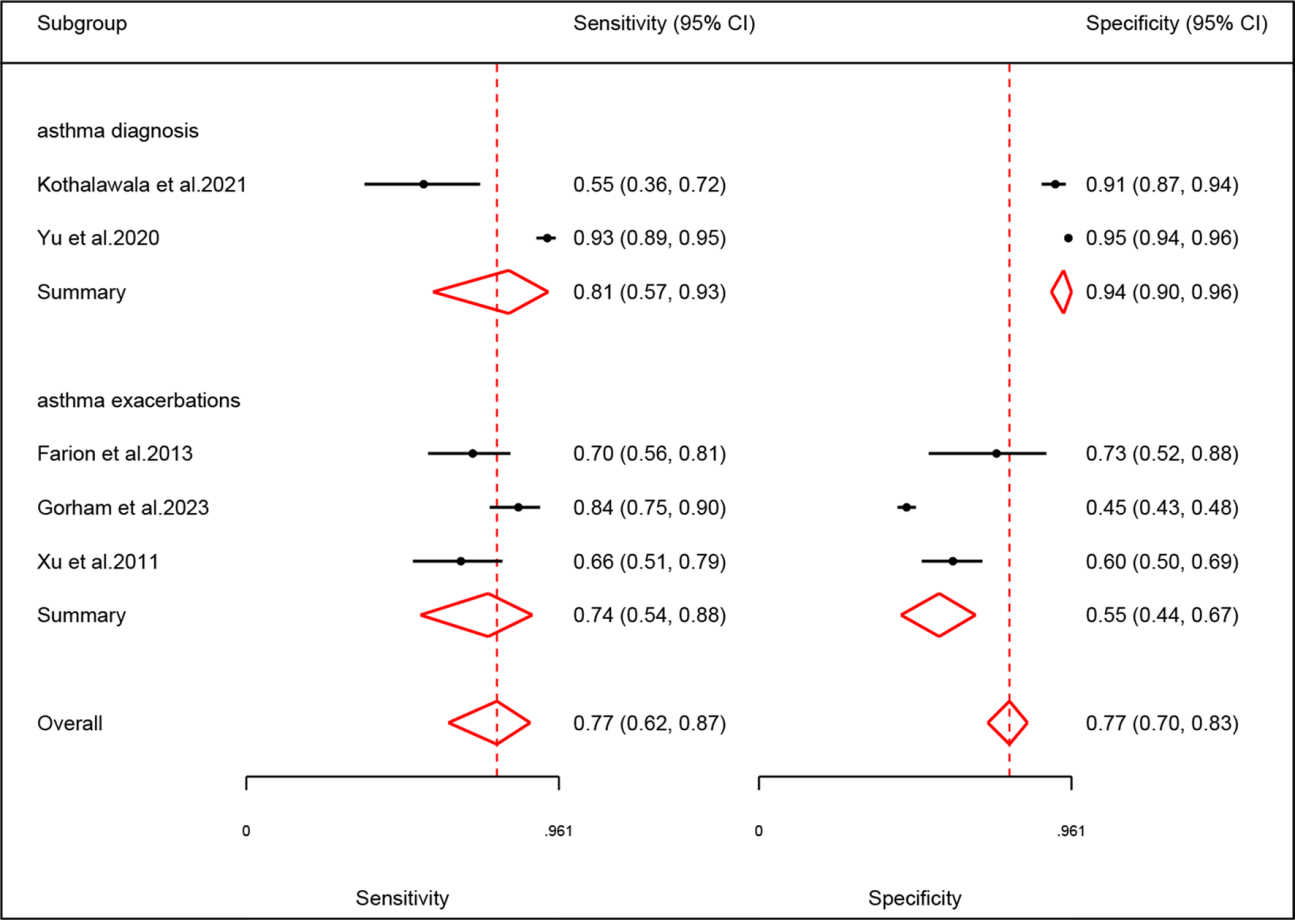
Forest plot depicting the external validation of AI models for asthma diagnosis and the prediction of acute exacerbations. The squares indicate sensitivity and specificity estimates from individual studies, with horizontal bars representing 95% confidence intervals. AI, artificial intelligence

For the diagnosis of asthma, the AI model had a sensitivity of 0.81 (95% CI: 0.57–0.93) and a specificity of 0.94 (95% CI: 0.90–0.96) (Fig. 7); for acute asthma exacerbations, the AI model had a sensitivity of 0.74 (95% CI: 0.54–0.88), and the specificity was 0.55 (95% CI: 0.44–0.67) (Fig. 7).

### Diagnostic performance of subgroup analysis in internal validation sets for AI in diagnosing asthma

In terms of study types, the sensitivity of the retrospective study and the prospective study was 0.91 (95% CI: 0.81-1.00) and 0.78 (95% CI: 0.53-1.00), respectively, and the differences were not statistically significant (*P* = 0.29). Furthermore, the specificity of the retrospective study and the prospective study was 0.89 (95% CI: 0.78–0.99) and 0.97 (95% CI: 0.94-1.00), respectively, and the differences were not statistically significant (*P* = 0.21) (Table 4).

Among the study source countries, the sensitivity levels for Asian and non-Asian regions were 0.90 (95% CI: 0.71-1.00) and 0.86 (95% CI: 0.73–0.99), respectively, and the differences were not statistically significant (*P* = 0.56). Furthermore, the specificities for Asian and non-Asian regions were 0.92 (95% CI: 0.78-1.00) and 0.94 (95% CI: 0.88-1.00), respectively, and the differences were not statistically significant (*P* = 0.99) (Table 4).

In AI models, the sensitivity of the DL subgroup and the ML subgroup was 0.99 (95% CI: 0.96-1.00) and 0.84 (95% CI: 0.72–0.96), respectively, and the specificity was 1.00 (95% CI: 1.00–1.00) and 0.92 (95% CI: 0.86–0.98), respectively (Table 4). Because the diagnostic DL subgroup contained only one study, these findings should be interpreted as exploratory and should not be taken as evidence that DL is superior to ML.

In the subgroup analysis stratified by whether data were reconstructed from ROC curves, the pooled sensitivity was 0.82 (95% CI, 0.48–1.00) for reconstructed data and 0.88 (95% CI, 0.76–0.99) for non-reconstructed data. The corresponding pooled specificities were 0.84 (95% CI, 0.56–1.00) and 0.94 (95% CI, 0.89–1.00), respectively. No statistically significant between-subgroup differences were observed for either sensitivity (*P* = 0.97) or specificity (*P* = 0.49) (Table 4).

Subgroup analysis based on data source revealed variations in model performance. Studies utilizing Electronic Health Record (EHR) data yielded a pooled sensitivity of 0.94 (95% CI: 0.87-1.00), while those using genomic data showed a pooled sensitivity of 0.32 (95% CI: 0.13–0.78), which was statistically significant (*P* < 0.001) (Supplementary Table 5, Table 4). Conversely, specificity was comparable between EHR-based studies (0.93, 95% CI: 0.83-1.00) and genomic studies (0.99, 95% CI: 0.94-1.00; *P* = 0.80) (Table 4).

### Publication bias

**Fig. 8:**
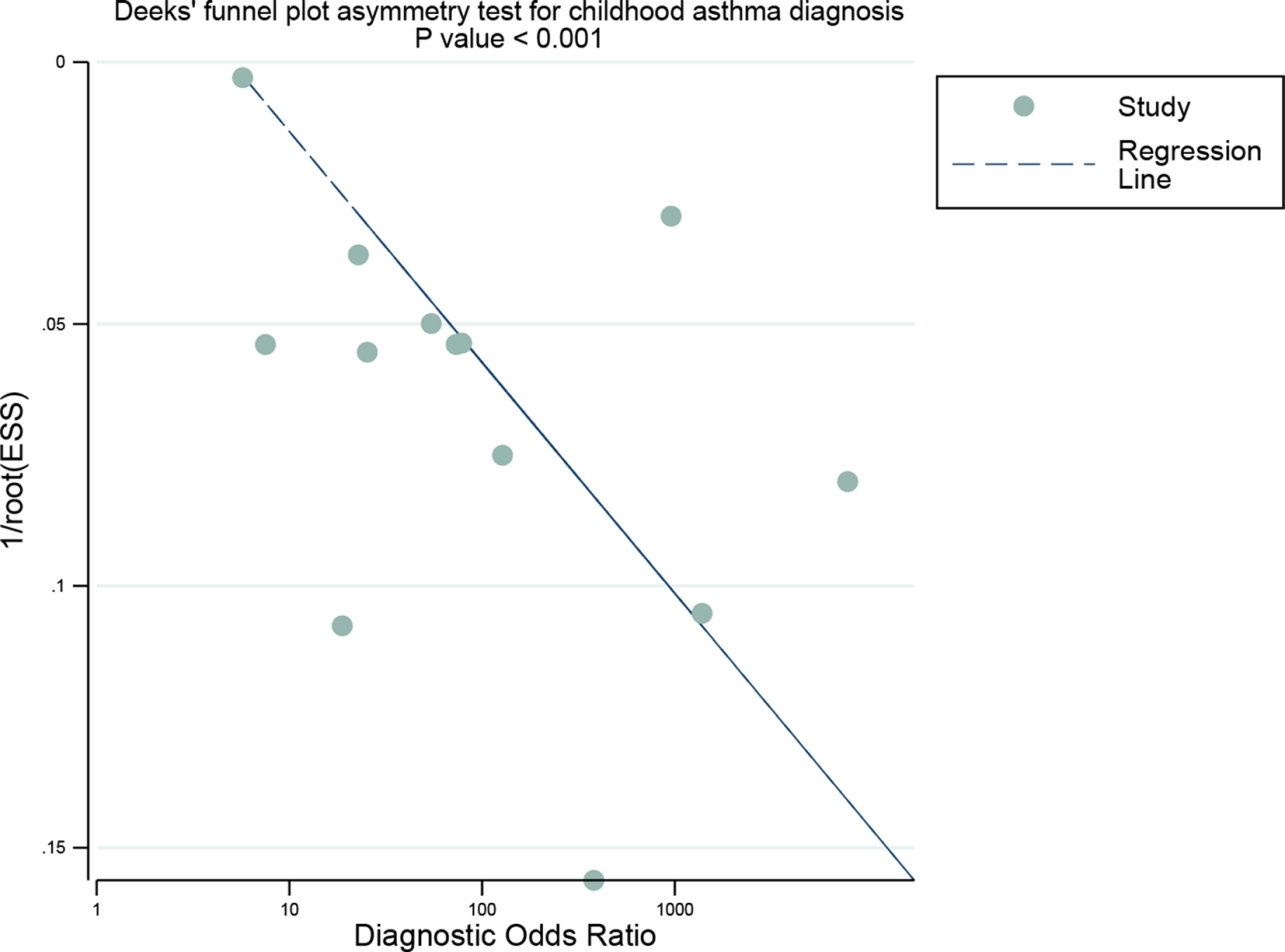
Deeks’ funnel plot asymmetry test was used to assess publication bias for AI-based models in the diagnosis of pediatric asthma in internal validation. A P-value of < 0.05 was deemed significant. AI, artificial intelligence

For asthma diagnosis, Deeks’ funnel plot asymmetry test indicated significant funnel plot asymmetry in the internal validation sets (*P* < 0.001; Fig. 8), suggesting possible publication bias or small-study effects. As prespecified in the Methods, no publication-bias test was performed for acute exacerbation prediction because fewer than ten studies were available, and no trim-and-fill adjustment was applied in this diagnostic accuracy meta-analysis.

### Sensitivity analysis

A sensitivity analysis excluding studies with high risk of bias or high applicability concerns was performed. For asthma diagnosis, the AI models had a sensitivity of 0.75 (95% CI: 0.38–0.94), specificity of 0.97 (95% CI: 0.74-1.00), and AUC of 0.95 (95% CI: 0.92–0.96) (Supplementary Fig. 1). A sensitivity analysis excluding studies with possible predictor-reference overlap was performed. For asthma diagnosis, the AI models had a sensitivity of 0.86 (95% CI: 0.55–0.97), specificity of 0.97 (95% CI: 0.84–0.99), and AUC of 0.97 (95% CI: 0.96–0.99) (Supplementary Fig. 2). Sensitivity analyses excluding Ahmadiankalati 2024 [19] and Hamad 2023 [22] were also conducted, as the former reported markedly divergent accuracy estimates and the latter had an extremely large sample size that may exert disproportionate influence on the pooled results, and both analyses yielded results consistent with the primary analysis (Supplementary Fig. 3).

## Discussion

This systematic review and meta-analysis suggests that AI-based models may have promising diagnostic performance for pediatric asthma, particularly in internal validation datasets. However, the high AUC and pooled sensitivity and specificity should be interpreted cautiously because the evidence base was characterized by very high heterogeneity, variable reference standards, possible publication bias, and limited external validation.

For acute asthma exacerbation prediction, AI models showed limited and inconsistent performance across validation settings. Internal validation showed only moderate performance, and external validation remained sparse. These findings indicate that exacerbation prediction models require further development, transparent reporting, and independent validation before clinical use. Given the very high residual heterogeneity and the small number of external validation datasets, the pooled estimates should be regarded as exploratory summary evidence rather than definitive evidence of clinical readiness.

The substantial heterogeneity across the included studies may have influenced the pooled sensitivity and specificity estimates. Potential sources of heterogeneity included study design, geographic region, AI method, reconstruction of data from ROC curves, and data source [36, 37]. The subgroup analysis by AI method suggested an apparently higher specificity in the DL subgroup; however, this subgroup included only one diagnostic study. Therefore, this finding should be considered hypothesis-generating rather than evidence of DL superiority over ML. Future comparative studies using consistent datasets and predefined model categories are needed before any conclusion can be made about the relative performance of DL and ML. The observed discrepancy in sensitivity between EHR and genomic data may reflect the clinical heterogeneity of asthma as a syndrome diagnosed using clinical features rather than a purely monogenic disease [38]. EHR contains rich phenotypic and symptomatic data that may align more closely with clinical diagnoses, whereas genomic data primarily reflects genetic predisposition rather than current disease activity [39]. At this level of heterogeneity, a single pooled estimate should be interpreted as an exploratory summary rather than a definitive estimate applicable to all settings.

In addition, the funnel plot asymmetry suggested by Deeks’ test warrants a cautious approach regarding the clinical implementation of these AI tools. Furthermore, this meta-analysis provides a broad evaluation of AI-based models in pediatric asthma diagnosis and exacerbation prediction, but it does not directly compare AI models with conventional regression-based prediction models. Therefore, the incremental value of AI over traditional approaches remains uncertain and should be examined in future studies.

A 2024 meta-analysis by Martina Votto et al. [10] examined ML algorithms in predicting hospitalizations and emergency department (ED) visits due to acute exacerbations in children, reporting a pooled AUC of 0.67 for predicting ED visits and a higher AUC of 0.79 for predicting hospitalizations. In comparison, our analysis revealed a sensitivity of 0.59, specificity of 0.79, and AUC of 0.68 in internal validation sets, and a sensitivity of 0.74 and specificity of 0.55 in external validation sets for AI models predicting acute exacerbations. The relatively lower diagnostic performance in our results compared to Martina Votto et al. may be attributed to their focus on AUC as the primary outcome, allowing for a larger number of included studies. Compared to their study, we assessed more outcomes, sensitivity and specificity, offering a more detailed evaluation [40].

Several limitations should be considered. First, most included studies relied on internal validation and small sample size, which may overestimate performance. Second, external validation was limited, reducing confidence in generalizability. Third, reference standards varied across studies and included specialist diagnosis, chart review, ICD-based surveillance definitions, parent-reported wheeze, and questionnaire-based definitions. This lack of a uniform gold standard may have contributed to heterogeneity and highlights the need for a standardized AI-ready definition of pediatric asthma. Fourth, some studies may have predictor-reference overlap, which could introduce target leakage. Fifth, selecting the best-performing model from each study may have overestimated performance. Sixth, some 2 × 2 tables were reconstructed rather than directly reported.

## Conclusion

AI-based models showed promising but preliminary diagnostic performance for pediatric asthma, while their ability to predict acute asthma exacerbations remained limited. The evidence is constrained by very high heterogeneity, possible publication bias, variable reference standards, limited external validation, and potential overestimation from internal validation. Future studies should adopt standardized AI-ready definitions of pediatric asthma and asthma exacerbation, compare AI with conventional models, and prioritize external validation across diverse geographic and clinical settings before clinical implementation.

## Supplementary Information

Below is the link to the electronic supplementary material.

Supplementary Material 1

## Data Availability

All data generated or analysed during this study are included in this published article and its supplementary information files.

## Declarations

### Generative AI and AI-assisted technologies in the writing process

During the preparation of this work, the authors used Sider in order to improve readability and language quality. After using this tool, the authors reviewed and edited the content as needed and take full responsibility for the content of the publication.
