# Supplementary material for: Artificial intelligence-based models in predicting acute exacerbations and diagnosing pediatric asthma: a systematic review and meta-analysis
Source: BMC Med Inform Decis Mak. 2026 May 29;26:289. doi: 10.1186/s12911-026-03599-7 (PMC13440168; doi:10.1186/s12911-026-03599-7)
Supplement: Supplementary file 1 [file 12911_2026_3599_MOESM1_ESM.docx]

### Supplementary Table 1. Preferred Reporting Items for Systematic Reviews and Meta-Analyses of Diagnostic Test Accuracy (PRISMA-DTA) Checklist.

| **TITLE/ABSTRACT** | | | **Reported on page #** |
| --- | --- | --- | --- |
| **Title** | 1 | Identify the report as a systematic review (+/−meta-analysis) of diagnostic test accuracy (DTA) studies | Page 1 |
| **Abstract** | 2 | See the PRISMA for Abstracts checklist. | Page 2 |
| **INTRODUCTION** | | |  |
| **Rationale** | 3 | Describe the rationale for the review in the context of what is already known | Pages 2-3 |
| **Clinical role of index test** | D1 | State the scientific and clinical background, including the intended use and clinical role of the index test, and if applicable, the rationale for minimally acceptable test accuracy (or minimum difference in accuracy for comparative design) | Pages 2-3 |
| **Objectives** | 4 | Provide an explicit statement of question(s) being addressed in terms of participants, index test(s), and target condition(s) | Page 3 |
| **METHODS** | | |  |
| **Protocol and**  **registration** | 5 | Indicate if a review protocol exists, if and where it can be accessed (e.g., Web address), and, if available, provide registration information including registration number. | Page 3 |
| **Eligibility criteria** | 6 | Specify study characteristics (participants, setting, index test(s), reference standard(s), target condition(s), and study design) and report characteristics (e.g., years considered, language, publication status) used as criteria for eligibility, giving rationale. | Page 3 |
| **Information sources** | 7 | Describe all information sources (e.g., databases with dates of coverage, contact with study authors to identify additional studies) in the search and date last searched. | Page 3 |
| **Search** | 8 | Present full search strategies for all electronic databases and other sources searched, including any limits used, such that they could be repeated. | Page 3; Supplementary Table 2 |
| **Selection process** | 9 | State the process for selecting studies (i.e., screening, eligibility, included in systematic review, and, if applicable, included in the meta-analysis). | Pages 3 and 5; Figure 1 |
| **Data collection process** | 10 | Describe method of data extraction from reports (e.g., piloted forms, independently, in duplicate) and any processes for obtaining and confirming data from investigators. | Page 3 |
| **Definitions for data extraction** | 11 | Provide definitions used in data extraction and classifications of target condition(s), index test(s), reference standard(s) and other characteristics (e.g., study design, clinical setting). | Pages 3-4; Tables 1-3 |
| **Risk of bias and**  **applicability** | 12 | Describe methods used for assessing risk of bias in individual studies and concerns regarding the applicability to the review question. | Page 3; Figure 2; Supplementary Tables 3-4 |
| **Diagnostic accuracy measures** | 13 | State the principal diagnostic accuracy measure(s) reported (e.g., sensitivity, specificity) and state the unit of assessment (e.g., per-patient, per-lesion). | Page 4 |
| **Synthesis of results** | 12 | Describe methods of handling data, combining results of studies and describing variability between studies. This could include, but is not limited to: a) handling of multiple definitions of target condition. b) handling of multiple thresholds of test positivity, c) handling multiple  index test readers, d) handling of indeterminate test results, e) grouping and comparing tests, f) handling of different reference standards | Page 4 |
| **Meta-analysis** | D2 | Report the statistical methods used for meta-analyses, if performed. | Page 4 |
| **Additional analyses** | 16 | Describe methods of additional analyses (e.g., sensitivity or subgroup analyses, meta-regression), if done, indicating which were pre-specified. | Page 4 |
| **RESULTS** | | |  |
| **Study selection** | 17 | Provide numbers of studies screened, assessed for eligibility, included in the review (and included in meta-analysis, if applicable) with reasons for exclusions at each stage, ideally with a flow diagram. | Page 5; Figure 1 |
| **Study characteristics** | 18 | For each included study provide citations and present key characteristics including: a) participant characteristics (presentation, prior testing), b) clinical setting, c) study design, d) target condition definition, e) index test, f) reference standard, g) sample size, h) funding sources | Page 5; Table 1 |
| **Risk of bias in studies** | 19 | Present evaluation of risk of bias and concerns regarding applicability for each study. | Page 5; Figure 2; Supplementary Table 3 |
| **Results of individual studies** | 20 | For each analysis in each study (e.g., unique combination of index test, reference standard, and positivity threshold) report 2x2 data (TP, FP, FN, TN) with estimates of diagnostic accuracy and confidence intervals, ideally with a forest or receiver operator characteristic (ROC) plot. | Pages 5 and 10-15; Tables 2-3; Figures 3-7 |
| **Synthesis of results** | 22 | Describe test accuracy, including variability; if meta-analysis was done, include results and confidence intervals. | Pages 5-6; Figures 3-7 |
| **Additional analysis** | 23 | Give results of additional analyses, if done (e.g., sensitivity or subgroup analyses, meta-regression; analysis of index test: failure rates, proportion of inconclusive results, adverse events). | Pages 5-6; Table 4 |
| **DISCUSSION** | | |  |
| **Summary of evidence** | 24 | Summarize the main findings including the strength of evidence. | Page 6 |
| **Limitations** | 25 | Discuss limitations from included studies (e.g., risk of bias and concerns regarding applicability) and from the review process (e.g., incomplete retrieval of identified research). | Page 6 |
| **Conclusions** | 26 | Provide a general interpretation of the results in the context of other evidence. Discuss implications for future research and clinical practice (e.g., the intended use and clinical role of the index test). | Page 6 |
| **FUNDING** | | |  |
| **Funding** | 26 | For the systematic review, describe the sources of funding and other support and the role of the funders. | Page 2 |

Salameh, Jean-Paul et al. “Preferred reporting items for systematic review and meta-analysis of diagnostic test accuracy studies (PRISMA-DTA): explanation, elaboration, and checklist.” *BMJ (Clinical research ed.)* vol. 370 m2632. 14 Aug. 2020, doi:10.1136/bmj.m2632

Supplementary Table 2. Search strategy in PubMed, Embase and Web of Science.

| **Database** | **Search strategy** |
| --- | --- |
| PubMed | ("Artificial Intelligence"[Mesh] OR "Machine Learning"[Mesh] OR "Deep Learning"[Mesh] OR "artificial intelligence"[Title/Abstract] OR "machine learning"[Title/Abstract] OR "deep learning"[Title/Abstract] OR "AI"[Title/Abstract] OR "ML"[Title/Abstract] OR "DL"[Title/Abstract]) AND ("Asthma"[Mesh] OR "asthma"[Title/Abstract]) AND ("Child"[Mesh] OR "Adolescent"[Mesh] OR "Pediatrics"[Mesh] OR "child*"[Title/Abstract] OR "pediatric*"[Title/Abstract] OR "paediatric*"[Title/Abstract] OR "adolescent*"[Title/Abstract]) AND ("Sensitivity and Specificity"[Mesh] OR "ROC Curve"[Mesh] OR "Predictive Value of Tests"[Mesh] OR “sensitivity”[Title/Abstract] OR “specificity”[Title/Abstract] OR "ROC curve"[Title/Abstract] OR “ROC”[Title/Abstract] OR “AUC”[Title/Abstract] OR "area under curve"[Title/Abstract] OR "c-index"[Title/Abstract] OR "predictive value*"[Title/Abstract]) |
| Embase | ('artificial intelligence'/exp OR 'machine learning'/exp OR 'deep learning'/exp OR 'artificial intelligence':ti,ab OR 'machine learning':ti,ab OR 'deep learning':ti,ab OR 'AI':ti,ab OR 'ML':ti,ab OR 'DL':ti,ab) AND ('asthma'/exp OR 'asthma':ti,ab) AND ('child'/exp OR 'adolescent'/exp OR 'pediatrics'/exp OR 'child*':ti,ab OR 'pediatric*':ti,ab OR 'paediatric*':ti,ab OR 'adolescent*':ti,ab) AND ('sensitivity and specificity'/exp OR 'roc curve'/exp OR 'predictive value'/exp OR 'sensitivity':ti,ab OR 'specificity':ti,ab OR 'roc curve':ti,ab OR 'roc':ti,ab OR 'auc':ti,ab OR 'area under curve':ti,ab OR 'c-index':ti,ab OR 'predictive value*':ti,ab) |
| Web of Science | (((TS=("artificial intelligence" OR "machine learning" OR "deep learning" OR “AI”)) AND TS=(“asthma”)) AND TS=(“child*” OR “pediatric*” OR “paediatric*” OR “adolescent*”)) AND TS=((“sensitivity” OR “specificity” OR "ROC curve" OR “ROC” OR “AUC” OR "area under curve" OR “c-index” OR “predictive value”)) |

Supplementary Table 3. Completed QUADAS-2 quality assessment for included studies.

| **Author** | **Risk of bias** | | | | **Applicability concerns** | | |
| --- | --- | --- | --- | --- | --- | --- | --- |
|  | **Patient**  **selection** | **Index**  **test** | **Reference standard** | **Analysis** | **Patient selection** | **Index test** | **Reference standard** |
| Afzal et al.2013 | L | H | L | L | L | L | L |
| Ahmadiankalati et al.2024 | L | L | L | L | L | L | L |
| Dexheimer et al.2007 | U | H | L | L | L | L | L |
| Filippo et al.2024 | H | L | L | L | L | L | L |
| Hamad et al.2023 | U | H | L | L | L | L | L |
| Kothalawala et al.2021 | L | L | L | L | L | L | L |
| Maduko et al.2007 | U | L | U | L | H | L | L |
| Pooja et al.2024 | L | L | L | L | L | L | L |
| Raciborski et al.2018 | U | L | L | L | L | L | L |
| Salih et al.2024 | L | L | L | L | L | L | L |
| Sanders et al.2006 | L | H | L | L | L | L | L |
| Tomita et al. 2004 | U | U | U | L | L | L | L |
| Yu et al.2020 | L | H | L | L | L | L | L |
| Farion et al.2013 | L | L | L | L | L | L | L |
| Gorham et al.2023 | U | L | L | L | L | L | L |
| Rezaeiahari et al.2024 | L | L | L | L | L | L | L |
| Wang et al.2019 | L | L | L | L | L | L | L |
| Xu et al.2011 | L | L | L | L | L | L | L |

L low risk; H high risk; U unclear risk.

Supplementary Table 4. Modified QUADAS-2 assessment tool with PROBAST-informed signaling questions.

| Domain | Modified QUADAS-2 signaling questions and applicability considerations |
| --- | --- |
| Patient selection | Were participants enrolled consecutively or randomly? Were inappropriate exclusions avoided? Did the population match children and adolescents aged 0-17 years with asthma or risk of asthma exacerbation? |
| Index test (AI algorithms) | Were predictors measured before or independently from the reference standard? Was possible predictor-reference overlap avoided? Was the validation strategy clearly reported? Was the selected model prespecified or transparently selected? |
| Reference standard | Was the reference standard likely to correctly classify asthma diagnosis or asthma exacerbation? Was the reference standard applied consistently and independently from the AI algorithm? |
| Analysis | Were TP, FP, FN, and TN directly reported or transparently reconstructed? Were missing data and small sample size considered? Was internal or external validation clearly defined? Was overfitting addressed? |
| Applicability concerns | Were patient selection, AI input data, and reference standards applicable to pediatric asthma diagnosis or exacerbation prediction in clinical practice? |

Supplementary Table 5. Specific data sources of included studies on childhood asthma diagnosis

| Predicting variables | Afzal et al., 2013 | Ahmadiankalati et al, 2024 | Dexheimer et al, 2007 | Filippo et al, 2024 | Hamad et al, 2023 | Kothalawala et al, 2021 | Maduko et al, 2007 | Pooja et al, 2024 | Raciborski et al, 2018 | Salih et al, 2024 | Sanders et al, 2006 | Tomita et al, 2004 | Yu et al, 2020 |  |
| --- | --- | --- | --- | --- | --- | --- | --- | --- | --- | --- | --- | --- | --- | --- |
| Electronic Health Records | + |  | + |  | + |  | + |  |  | + | + |  | + |  |
| Genomic Data |  | + |  |  |  |  |  |  |  |  |  | + |  |  |
| Medical Imaging Data |  |  |  | + |  |  |  |  |  |  |  |  |  |  |
| Clinical Text Data | + |  | + |  |  |  |  |  |  |  |  |  |  |  |
| Other |  | + |  |  |  | + |  | + | + |  |  |  |  |  |

Supplementary Figure 1. Sensitivity analysis of AI model performance for asthma diagnosis after excluding studies with high risk of bias.

Supplementary Figure 2. Sensitivity analysis of AI model performance for asthma diagnosis after excluding studies with potential predictor-reference overlap.

Supplementary Figure 3.Sensitivity analysis excluding influential studies (Ahmadiankalati 2024 and Hamad 2023) to assess the robustness of pooled estimates. (a). Exclusion of Ahmadiankalati 2024. (b). Exclusion of Hamad 2023.
